# Health and Ethical Consequences of Outsourcing Pivotal Clinical Trials to Latin America: A Cross-Sectional, Descriptive Study

**DOI:** 10.1371/journal.pone.0157756

**Published:** 2016-06-23

**Authors:** Núria Homedes, Antonio Ugalde

**Affiliations:** 1 Management Policy and Community Health, School of Public Health, University of Texas, El Paso, Texas, United States of America; 2 Department of International Health, Georgetown University, Washington DC, United States of America; 3 Department of Sociology, University of Texas, Austin, Texas, United States of America; Hvidovre Hospital, DENMARK

## Abstract

**Introduction:**

The implications of conducting clinical trials in low and middle income countries on the financial accessibility and safety of the pharmaceutical products available in those markets have not been studied. Regulatory practices and ethical declarations lead to the commercialization of the new products, referred to as New Molecular Entities (NMEs), in the countries where tested as soon as they are approved in high surveillance countries. Patients and patients’ associations use the Latin American courts to access new and expensive treatments, regardless of their safety profile and therapeutic value.

**Design and Objectives:**

Cross-sectional, descriptive study. To determine the therapeutic value and safety profile of the NMEs approved by the Food and Drug Administration (FDA) in 2011 and 2012 that had been tested in Latin America, and the implications of their market approval for the pharmaceutical budgets in the countries where tested.

**Setting:**

Latin America.

**Measures:**

To assess the therapeutic value and safety of the NMEs commercialized in the different countries we used f independent drug bulletins. The prices of the NMEs for the consumers were obtained from the pharmaceutical price observatories of the countries were the medicines had been tested. If the price was not available in the observatories, it was obtained from pharmaceutical distributors. We used the countries’ minimum wage and per capita income to calculate the financial accessibility of a course of treatment with the NMEs.

**Results:**

We found that 33 NMEs approved by the FDA in 2011 and 2012 have been tested in Latin America. Of these, 26 had been evaluated by independent drug bulletins and only five were found to add some value to a subset of patients and had significant side-effects. The pharmaceutical prices were very high, varied widely across countries and were unrelated to the countries’ income per capita or minimum wage.

**Conclusion:**

The implementation of clinical trials in Latin America results in the commercialization of medicines with questionable safety profiles and limited therapeutic value, putting patients at risk and causing budgetary strains in pharmaceutical budgets.

## Introduction

The trend in outsourcing clinical trials (CTs) to low and middle income countries (LMICs) has been well documented. Shortening the duration of the clinical trials has been identified as one of the major strategies to expand the duration of inventors’ monopoly-power. To that effect, an increasing number of clinical-trial participants are recruited in LMICs (or host countries) [1, 2], where patients are more easily recruited and retained, allowing the pharmaceutical industry to expedite the completion of the CTs and the attainment of marketing approval for the new molecular entities, thus maximizing the NMEs’ market-exclusivity period [3]. The inability to recruit enough research participants in high-income countries [4] and the few regulatory hurdles in LMICs reinforce this tendency.

There has been little scrutiny of the consequences that conducting CTs has on the availability, appropriate and safe use of the new pharmaceuticals, as well as on the health budgets of the host countries. International ethical declarations require that approved NMEs be made available to the populations in which they have been tested [5]. The Latin American regulatory agencies base their marketing decisions on the actions taken by their counterparts in “high sanitary surveillance countries” (United States, Japan, Australia, selected individual countries in Europe and the European Medicines Agency). In other words, once one of these agencies approves the commercialization of a NME, the drug should be made available in the countries where it had been tested.

Additionally, Latin American patients and patient groups are increasingly using the judiciary system to exercise their constitutional right to health, including access to new and expensive pharmaceuticals. Vargas-Pélaez et al [6] conducted a scoping study of the literature on lawsuits for access to medicines and health services. They identified 65 articles, and 80% of them involved a Latin American country (68% Brazil, 9% Colombia and 3% Argentina). The Latin American authors cited in this study mentioned that in some cases the courts decide without taking into consideration the evidence of drug efficacy and safety or the appropriateness of the treatment for a particular patient, possibly putting the plaintiff at risk of adverse effects and drug misuse. Moreover, some authors asserted that the pharmaceutical industry was interested in promoting access to medicines through the courts, because it resulted in the inclusion of medicines in the public formularies that might be useful for only a small group of patients rather than the needs of society.

The final result of this ethical principle and regulatory practice is that those countries where the NMEs have been tested have to cover the costs of the NMEs, regardless of their safety profile and whether they offer any advantage over cheaper existing treatments.

While the magnitude of the financial impact will differ across countries and will in part depend on the sales price of the NMEs in each country, public coverage of these new and expensive NMEs will strain the public pharmaceutical budgets.

The health consequences of outsourcing CTs have been off the radar of researchers, possibly because it is assumed that the regulatory agencies of “high sanitary surveillance countries” only allow the commercialization of products that are safe and effective, and what is available to the residents of high income countries ideally should also be offered to the residents of less prosperous countries, especially if they have contributed to their development.

This is the first study that questions the health benefits of the CTs implemented in Latin America not only for CT subjects but for the health systems and residents of those countries.

Using information on pricing and value of the NMEs approved by the FDA in 2011 and 2012 that were tested in Latin America, this article analyzes the health, financial and some ethical consequences of outsourcing CTs to the region.

## Methods

This is a cross-sectional, descriptive study. The objectives were: (1) to determine the therapeutic value and safety of the NMEs approved by the FDA and tested in Latin America; and (2) to assess the financial accessibility of the NMEs in the countries where they had been tested. The list of NMEs approved by the FDA in 2011 and 2012 was obtained from FDA publications [7, 8]. The FDA’s medical reviews of the NMEs, included in the FDA’s drug approval history, provided the names of the countries where the CTs had been conducted. The drug approval histories can be found in the Drugs@FDA website [9]. If the medical reviews fail to mention the countries where the pivotal trials were conducted, we contacted the trial sponsors. It is possible that a few Latin American CTs included in our study were non-pivotal trials.

Data bases from two reputable independent drug bulletins, namely Prescrire (France) and the Health Research Group of Public Citizen (HRG) in the United States were consulted for evidence of the added therapeutic value of the NMEs to existing treatments. Prescrire often included information from other independent bulletins. The assessment of cancer treatments was supplemented with information from Fojo et al.[10].

The price of the unit dose of each product was obtained from the countries’ price observatories, which report the maximum price to consumers (Brazil, Mexico) or the observed consumer prices (Argentina, Chile, Colombia, Ecuador, Peru). In a few cases, when the information was not available in the observatories, local pharmaceutical experts obtained it from local distributors. The quantities needed to complete a course or a year of treatment, in the case of chronic conditions, were calculated by the authors using the recommendations included in the FDA-approved product label. We assessed the cost of a course of treatment with each NME in the countries where tested in absolute value, and as a proportion of the countries’ monthly minimum wage and monthly per capita income. The pricing information was gathered between August 25th and September 20th, 2014. (For additional information on pricing sources and the methodology to determine financial accessibility see Homedes and Ugalde [11]).

## Findings

The 33 NMEs approved by the FDA in 2011 and 2012 and the Latin American countries where they were tested are listed in Table 1.

**Table 1:** Products approved by the United States Food and Drug Administration in 2011 and 2012 that were tested in pivotal trials in Latin America.

Non-proprietary name	Commercial name	Pharmaceutical company	Countries where pivotal clinical trials were conducted
Aclidium bromide	Tudorza Pressair/Eklaire Genuari	Forest/Almirall	Peru
Aflibercept	Eylea/Eylia	Bayer	Argentina, Brazil, Chile, Colombia, Mexico
Apixaban	Eliquis/Elicuis	BMS	Argentina, Brazil, Chile, Colombia, Mexico, Peru
Axitinib	Inlyta	Pfizer	Brazil
Azilsartan Medoxonil	Edarbi	Takeda	Argentina, Chile, Mexico, Peru
Bedaquiline	Sirturo	Janssen	Brazil
Belatacept	Nulojix	BMS	Argentina, Brazil, Chile, Mexico,
Belimumab	Benlysta	GSK	Argentina, Brazil, Chile, Colombia, Costa Rica, Mexico, Peru
Bosutinib	Bosulif	Pfizer	Argentina, Brazil, Chile, Colombia, Mexico, Peru
Cabozantinib	Cometriq	Exelixis/Sobi	Brazil, Chile, Peru
Crizotinib	Xalkori	Pfizer	Brazil
Elvitegravir,cobicistat, emtricitabine,tenofovir disoproxil fumarate	Stribild	Gilead	Mexico
Enzalutamide	Xtandi	Raffo/Astellas	Argentina, Chile
Ezogabine	Potiga	GSK	Argentina, Brazil, Mexico
Indacaterol maleate	Arcapta Neohaler/Onbrize	Novartis	Argentina, Chile, Colombia, Ecuador, Peru
Ipilimumab	Yerboy/Yervoy	BMS	Argentina, Brazil, Chile, Peru
Linagliptin	Tradjenta	Boehringer	Argentina, Mexico
Lucinactant	Surfaxin	Discovery	Brazil, Chile, Ecuador, Mexico, Panama, Uruguay
Pasireotide	Signifor	Novartis	Argentina, Brazil, Mexico
Perampanel	Fycompa	Eisai	Argentina, Chile, Mexico
Pertuzumab	Perjeta	Genentech/Roche	Brazil, Mexico, Peru
Regorafenib	Stivarga	Bayer	Argentina, Brazil
Rilpivirine	Edurant	Janssen	Argentina, Brazil, Chile, Costa Rica, Mexico, Panama
Rivaroxavan	Xarelto	Bayer/Janssen	Argentina, Brazil, Chile, Colombia, Mexico, Peru, Venezuela
Roflumilast	Daliresp/Daxas	Forest/Takeda	Brazil
Taliglucerase alfa	Elelyso/Uplyso	Pfizer	Chile
Tbo-filgastrim	Neutroval/Granix	Teva	Brazil, Chile
Telaprevir	Incivek	Janssen/Vertex	Argentina, Brazil
Teriflunomide	Aubagio	Genzyme	Chile, Colombia
Ticagrelor	Brilinta	AstraZeneca	Argentina, Brazil, Mexico,
Tofacitinib	Xeljan	Pfizer	Argentina, Brazil, Chile, Colombia, Costa Rica, Mexico, Peru, Dominican Republic, Venezuela
Vandetanib	Caprelsa	AstraZeneca	Argentina, Brazil, Mexico
Ziv-aflibercept	Zaltrap	Sanofi	Argentina, Brazil, Chile, Colombia, Mexico

### Therapeutic value and safety of the NMEs

Prescrire and/or HRG evaluated 26 of the 33 NMEs included in this study, and determined that 21 of the 26 (80%) offered no therapeutic advantage over existing treatments, had significant side effects and advised against the use of ten of them (See Table 2). According to these sources and the independent bulletins cited by Prescrire, the remaining five products (crizotinib, enzalutamide, ipilimumab, pasireotide, and telaprevir) could offer some advantage to a subset of patients, but the risk-benefit ratio was still uncertain. Only three of these five products were available in the countries where tested.

**Table 2:** Clinical relevance of NMEs approved by the FDA in 2011 and 2012 according to independent drug bulletins.

NME and indication	Assessment by independent drug bulletins
**Aclidinium bromide** [12] Patients with COPD	**Revue Prescrire.** Nothing new. Not better than existing treatments. Same cardiovascular adverse effects than others in its class
	**Arznei-Telegramm** (Germany). Do not recommend it. There is a need for more studies comparing it to other long-term bronchodilators. Long-term efficacy data and side-effects need to be better understood.
	**Drugs and Therapeutics Bulletin** (United Kingdom). Need to compare to other bronchodilators in phase III studies. Similar effects than placebo in terms of episodes that required the use of antibiotics, corticosteroids, hospitalization.
	**Gebu** (Netherlands). There is no information proving that it improves prognosis or limits exacerbations. No therapeutic advance. Do not use
**Aflibercept** [13] To treat patients with wet age-related macular degeneration (AMD)	**Revue Prescrire**. Does not add value to existing treatment with ranibizumab—measured in terms of efficacy, side-effects or ease of administration.
	**Medical Letter** (USA). Same efficacy as ranibizumab, and has not been tested against bevacizumab, cheaper and same efficacy.
	**Australian Prescriber** (Australia). Adverse events are similar to those of ranibizumab
**Apixaban** To reduce the risk of stroke and dangerous blood clots in patients with atrial fibrillation that is not caused by a heart valve problem.	**Revue Prescrire.** Not demonstrated to be better than warfarin (severe cases) or warfarine or aspirin (mild cases). Has not been compared to dabigatran. Poor evidence and there is no antidote [14].
	**Worst Pills, Best Pills.** We recommend that patients not use this drug for seven years after its approval date, because it does not represent a clear therapeutic breakthrough over the existing drug, warfarin [15].
**Bedaquiline** [16] To treat Multidrug resistant TB	**Worst Pills, Best Pills.** Do not use. Those receiving the drug were five times more likely to die than those receiving a placebo. Instead of looking into this more carefully, the FDA approved the drug with the warning: “In one clinical trial, more deaths were seen in people who were treated with Sirturo compared to people who did not receive Sirturo.
**Belatacept** [17] To prevent acute rejection in adult patients who have had a kidney transplant (10 mg per Kg per treatment).	**Revue Prescrire**. Nothing new. Not more effective and it is not less nephrotoxic in the long run. Adverse effects (lymphoma and infections) appear to be more frequent with belatacept. It is better to use cyclosporine.
	**Medical Letter** (USA). Same efficacy as cyclosporine after one year. It has not been compared to tracolimus. Has side effects—like lymphoma and serious infections. Need more long-term data
	**Arzneimittelkommission der deutschen Ärzteschaft** (Germany). Even though there are more kidney rejections, overall survival is similar to that of patients treated with cyclosporine. Was not evaluated against standard treatment. More comparative and long-term data are needed
**Belimumab** [18] For patients with active, autoantibody-positive lupus who are receiving standard therapy, including corticosteroids, antimalarials, immunosuppressives, and nonsteroidal anti-inflammatory drugs.	**Revue Prescrire.** Nothing new. When added to standard treatment, there is a small increase in the number of patients who respond to the treatment but it exposes the users to allergic reactions that can be severe, as well as to risks of cancer and infections that are not well defined. Do not complicate the treatment adding belimumab.
	**Medical Letter** (USA). Small reduction in the activity of lupus, appears to decrease the consumption of corticosteroids. Has not been studied in patients with renal problems linked to lupus or with severe problems in the central nervous system.
	**Info från Läkemedelsverket** (Sweden). Severe adverse drug reactions. The long term consequences are unknown.
**Bosutinib** [19] For adults with Philadelphia chromosome–positive chronic myelogenous leukemia who no longer benefit or tolerate other treatment.	**Revue Prescrire**. Could offer some benefits but in exchange for serious adverse events. Uncertain Risk-Benefit ratio. Best to use it only for research until side effects are better known.
**Crizotinib** [20] To treat lung cancer, non-small cell carcinoma, after other chemotherapies have failed.	**Revue Prescrire**.The benefit-risk ratio is uncertain. Probably an extra 8 months of life. However, the claim is made on radiology findings and there is no information on global survival. Need to have more studies
	**Medical Letter** (USA). Has prolonged life in 4–5% of lung cancer patients. The effect on overall survival is unknown
	**Arzneimittelkommission der deutschen Ärzteschaft** (Germany). Side effects are poorly evaluated. Need information on patients with liver or renal failure and older patients. Efficacy and safety evaluations are incomplete. Current information is encouraging, but comparative trials with palliative and other treatments have not been completed.
**Elvitegravir, cobicistat, emtricitabine, tenofovir, (Stribild)** [21] To treat HIV in adults who have never taken HIV medicines before.	**Revue Prescrire.** In the adult population, not better than other available combined-treatments in terms of convenience of administration, effectiveness or adverse events
	**Medical Letter** (USA). Can be useful in people who are HIV positive but have never received treatment. Cannot be used in patients with renal failure, has multiple drug interactions.
	**Der Arzneimittelbrief** (Germany). Can be an alternative. Need to know more about adverse events.
**Enzalutamide** [22] Treatment of patients with castration-refractory prostate cancer	**Revue Prescrire.** Similar to abitaterone. Can be useful if the patient cannot be treated with abiraterone (patients with cardiac or liver problems). Be mindful of drug interactions and seizures
	**Medical Letter** (USA). Second hormonal treatment that can increase survival on patients treated with docetaxel. No head-to-head studies comparing with abiraterone
	**Der Arzneimittelbrief** (Germany). Proven to increase survival by 4.8 months when compared with placebo. Need to pay attention to adverse effects and drug interactions
**Indacaterol maleate 75μg** [23] Long term maintenance of airflow obstruction in chronic obstructive pulmonary disease.	**Worst Pills, Best Pills.** Do not use. The FDA should have not approved the 75 μg dosage form, lower dosage had same effects. No advantages over other bronchodilators → does not deserve approval.
**Ipilimumab** [24] To treat patients with late-stage (metastatic) melanoma.	**Revue Prescrire**. Need more studies to evaluate benefit-risk ratio. A clinical trial with a questionable design showed an increase in overall survival, but there are serious adverse reactions which can compromise the quality of life.
	**Medical Letter** (USA). Capable of increasing life expectancy in patients with melanoma that cannot be surgically removed or has metastasized. Serious side effects.
	**Australian Prescriber** (Australia). More studies need to be done because patients with brain metastasis have been excluded from the studies.
	**Arznei-Teleg**ramm (Germany). In comparison with an experimental vaccine, ipilimumab increases life expectancy by a few months. The benefits are uncertain. 20% of the patients suffer serious adverse events, and 3.1% of the patients die. Too expensive, more Є100.000. We do not recommend its use
**Linagliptin** [25, 26] An adjunct to diet and exercise to improve glycemic control in adults with type 2 diabetes mellitus.	**Revue Prescrire**. No evidence of proven efficacy on diabetes complications. Not more effective than other gliptines. Adverse effects are severe, not worth using it
	**Medical Letter** (USA). The long term effects are unknown.
	**Australian Prescriber** (Australia). Adverse events: muscle-squeletal problems, high blood pressure, headaches, high level of triglycerides and uric acid, allergies and pancreatitis.
	**Pharma Selecta** (Netherlands). More studies are needed
	**Institut for rationel farmakoterapi** (Denmark). None of the clinical trials has compared linagliptine with other treatments of the same group. Few patients 75 years of age and over.
	**Worst Pills, Best Pills**. Do not use [25].
**Pasireotide** [27] To treat Cushing’s disease patients who cannot be helped through surgery.	**Revue Prescrire**. Possibly effective in 25% of the patients, but has many adverse effects, some of which are severe (hyperglycemia, gallbladder stones, diarrhea, nausea, prolongation of QT, bradycardia, hypothyroidism, low levels of cortisone etc). Only when there is no other treatment and the surgery has failed
	**Info från Läkemedelsverket** (Sweden). Only for those who cannot or do not want to have surgery, or when surgery has been insufficient. Good responders are easy to identify (during the second month of treatment) and for the non-respondents the treatment should be interrupted.
**Perampanel** [28] Adjunctive treatment of partial-onset seizures in epileptics aged >11 years	**Revue Prescrire.** No demonstrated added value. Adverse effects need to be better documented (cardiac toxicity, impact on growth).
**Pertuzumab** [29] To treat patients with HER2-positive late-stage (metastatic) breast cancer.	**Revue Prescrire.** The benefit-risk ratio is not well-known. Increases global survival of women with metastasis of breast cancer or with local recidivated cancer. Pertuzumab is added to trastuzumab+docetaxel; and it increases side effects. It should only be used in clinical trials
	**Australian Prescriber** (Australia). Appears to increase survival without worsening (progression) the condition of HER-2 positive women with metastasis of breast cancer.
	**Medical Letter** (USA). Same as Australian Prescriber but adds that the effect on overall survival has not been determined.
	**Der Arzneimittelbrief** (Germany). Adds 6.1 months of progression-free survival compared to placebo. Women participating in the trial were not representative of patients with this health problem. Considers that there is insufficient information to recommend its commercialization
	**Arzneimittelkommission der deutschen Ärzteschaft** (Germany). Serious Adverse effects more frequent in women treated (35.6%) than in placebo group (28%). The women studied are different than the typical patient population. Therefore the benefits are uncertain, especially in older women, with a more serious disease or previously treated.
	**Info från Läkemedelsverket** (Sweden). More adverse events, women with cardiac risks not included in the study
**Regorafenib** [30] To treat patients with colorectal cancer that has progressed after treatment and spread to other parts of the body.	**Revue Prescrire**. Appears to increase overall survival by several weeks (6.4 months with regorafenib, 5 months with placebo) in certain types of patients with metastatic colon cancer, in good condition, after several treatments. Many adverse events (40% of the patients), some of them serious, even deadly. Need more studies. Until then symptomatic treatment.
	**Medical Letter** (USA). Can improve free-progression survival in patients with metastasis of colon cancer or of local cancer that has already been treated. Adverse events in 50% of the patients.
**Rilpivirine** [31] For the treatment of HIV-1 infection in adults who have never taken HIV therapy.	**Revue Prescrire.** Not more effective than efarivenz. Rilpivirine causes more crossed resistances and it does not have less adverse events. Stay with Efavirenz
	**Medical Letter** (USA). Appears to be as effective as efarivenz in HIV positives not treated with antirretrovirals and could have less adverse effects. But the development of resistance and virus failures is more frequent with rilpivirine. The development of resistance to rilpivirine could lead to crossed resistances with other products.
	**Der Arzneimittelbrief** (Germany). An option with less side effects, but need to know more about resistance
	**Info från Läkemedelsverket** (Sweden). The development of resistance occurs more frequently with rilvipirine than with other ARVs. The development of resistance to rilpivirine could lead to crossed resistances with other products.
**Rivaroxavan** To reduce the risk of blood clots, deep vein thrombosis, and pulmonary embolism after knee or hip replacement.	**Revue Prescrire**. Not better than enoxaparine [32].
	**Worst Pills, Best Pills**. We recommended that patients do not use rivaroxavan for seven years after its approval date. It does not represent a clear therapeutic breakthrough over the existing drug, warfarin (Coumadin, Jantoven, Athrombin) [15].
**Roflumilast** [33]To decrease the frequency of flare-ups (exacerbations) or worsening of symptoms from severe chronic obstructive pulmonary disease (COPD).	**Revue Prescrire**. Better not to use it. Serious adverse events.
	**Medical Letter** (USA): offers some advantages, but due to side effects it is best to limit its use for people who do not respond to other treatments
	**Agence canadienne des medicaments et des technologies de la santé** (Canada). Minimum clinical improvements and too many side effects. Clinical trial data invalidated due to deviations of protocol and lack of data on important aspects of how patients evaluate the treatment
	**Arznei-Telegramm** (Germany). Too many side effects. Recommend not to use it.
	**Arzneimittelkommission der deutschen Ärzteschaft** (Germany). Efficacy has not been evaluated against reference treatments.
	**Pharma Selecta** (Netherlands): No information on long-term effects. Too many adverse events. Limited role on patients with severe COPD.
	**Navarra Salud** (Spain). Doubtful efficacy. Adverse events worrisome. Do not use
	**Dialogo Sui Farmaci** (Italy). Moderate efficacy, insufficient information about safety profile. Do not use.
	**Gebu** (Netherlands). Efficacy and safety insufficiently documented. Do not use.
	**Institut for rationel farm**akoterapi (Denmark). It has not been studied in comparison to standard treatment
**Telaprevir** [34] For certain adults with chronic hepatitis C.	**Revue Prescrire**. Might be indicated in certain patients, after they have tried boceprevir, longer studies are necessary with close monitoring of adverse events.
**Teriflunomide [**35, 36**]**, Multiple sclerosis	**Revue Prescrire**. Leflunomide was authorized in 1999. Teriflunomide is the main metabolite of leflunamide and its adverse effects should be the same. No demonstrated effect in improving or delaying the evolution of the problems. Better not to use it, and use interferon-beta.
	**Arznei-Telegramm** (Germany): No advantage
	**Pharma Sele**cta (Netherlands). Easy administration (oral). Adverse events. Little experience in multiple sclerosis (good experience in rheumatoid arthritis)
	**Info från Läkemedelsverket** (Sweden). Do not use in multiple sclerosis.
	**Drugs and Therapeutics Bulletin** (United Kingdom). Not better than other treatments
	**Australian Prescriber** (Australia). Not all patients benefit and the majority suffer adverse events. Benefits are modest and need to be balanced with side-effects.
**Ticagrelor** [37] To reduce cardiovascular death and heart attack in patients with acute coronary syndromes (ACS).	**Revue Prescrire.** Has not decreased mortality compared to clopidrogel. Has more adverse events. It is better to use clopidrogel associated with aspirin or just copidrogel.
	**Arznei-Telegramm** (Germany). Could be better than clopidrogel but seven times more expensive.
	**Arzneimittelkommission der deutschen Är**zteschaft (Germany). Better for some patients, worse for others. In general not better than clopidrogel
	**Der Arzneimittelbrief** (Germany). Risk/benefit ratio insufficiently evaluated
	**Info från Läkemedelsverket** (Sweden). Do not use for more than one year because the therapeutic experience is so far limited
	**Institut for rationel farmakoterapi** (Denmark). May have better outcomes, serious side effects
**Vandetanib** [38] Late-stage medullary thyroid cancer in adults, ineligible for surgery whose disease is growing or causing symptoms.	**Revue Prescrire**. No proven impact on survival in patients with metastatic or inoperable medullary thyroid cancer. Serious adverse events. More dangerous than beneficial.
**Ziv-aflibercept** [39] Colon cancer with metastasis.	**Revue Prescrire**. Aflibercept does not offer advantages over bevacizumab. Both products might add a few weeks and have very serious side effects (including death). Better not to use it
	**Medical Letter** (USA). Serious adverse events, but most of them are also present in patients treated with bevacizumab.
	**Arzneimittelkommission der deutschen Ärzteschaf**t (Germany). Combined with Folfire it has been associated with a moderate increase in survival than placebo, but it has shown more side effects. Risk-Benefit ration unclear
	**Info från Läkemedelsverket** (Sweden). Severe adverse events

Note: Neither Prescrire or the HRG of Public Citizen had evaluated azilsartan medoxonil, taliglucerasa alfa, tofacitinib, cabozantinib, ezogabine, lucinactant, tbo-filgastrim.

Of the 33 products included in our study, eight (25%) were included in Fojo et al’s evaluation [10]. Only one of them (enzalutamide) increased overall survival significantly: (by 4.8 months) in patients with castration-refractory prostate cancer); four increased the progression-free survival period (vandetanib, pertuzumab, carbozantinib, crizotinib), two NMEs did not fulfill the American Society of Clinical Oncology (ASCO) criteria to determine clinical relevance (ziv-aflibercept, regorafenib), and the authors were uncertain about ipilimumab (See Table 3).

**Table 3:** Efficacy of oncological treatments approved by the FDA in 2011 and 2012 as evaluated by Fojo and collaborators (2014).

NME	Indication	Gain in months		Would have met ASCO Criteria
		Progression-free survival (months)	Overall survival (months)	
Ipilimumab	First Line melanoma	0	2.1	Uncertain
Vandetanib	Advanced medullary thyroid carcinoma	11.1 (estimated)	NA	Yes
Pertuzumab	HER 2- positive breast cancer	6.1	NA	Yes
Ziv-aflibercept	Second line metastatic colorrectal cancer with FOLFIRI	2.2	1.44	No
Enzalutamide	Second line, castration-refractory prostate cancer	NA	4.8	Yes
Regorafenib	Metastatic colorrectal cancer	0.3	1.4	No
Carbozantinib	Advanced medullary thyroid carcinoma	7.2	NA	Yes
Crizotinib	Non-small cell lung cancer expressing ALK gene	4.7	NA	Yes

Source: Elaborated by authors from Fojo et al. [10]

In contrast with the methodology used by the independent drug bulletins mentioned above, Fojo et al. [10] assessed the value of each NME without comparing it with other treatment options. Two NMEs that qualified as useful in their publication (vandetanib and pertuzumab) were questioned by the independent drug bulletins. Vandetanib was considered more dangerous than beneficial, and the benefit-risk ratio of pertuzumab was judged to be insufficiently known. While Australian Prescriber and Medical Letter thought that it appeared to increase survival without worsening the condition of HER-2 positive women with metastasis of breast cancer, Medical Letter thought that the effect on overall survival had not been determined and others considered that it increased the side-effects, the benefits were uncertain, and there was insufficient information to recommend its commercialization. At a price of more than US$50,000 (pertuzumab) and US$100,000 (vandetanib) per treatment in Brazil and US$200,000 in Argentina (vandetanib) these NMEs are not affordable.

### Price of the NMEs in the Latin American countries where tested

As reported in a previous article by Homedes and Ugalde [11], two years after receiving market authorization in the United States, 12 of the 33 NMEs had not been registered or marketed in the Latin American countries where they had been tested. We obtained the prices of 18 of the remaining 21 NMEs and they are displayed in Table 4.

**Table 4:** Price of medicines by countries where tested, by number of months of income needed to pay for a course of treatment or a year of treatment for chronic conditions by monthly minimum wage (MMW), monthly income per capita (MIPC)) in US$. (All MNW and MIPC figures above 3 months have been rounded)^a^.

	Argentina	Brazil^b^	Chile	Colombia	Mexico	Peru
**GDP in purchasing power parity (PPP)** (World Bank, 2014)	12.568^c^	15,110	21,980	12,743	16,287	11,438
**NME and Dosage**						
**Aflibercept,** 8 injections per year						
Price	30,410	15,259	10,882	10,122	NI	--
MMW	58	46	28	33	NI	--
MIPC	25	16	8	16	NI	--
**Apixaban,** Two, 5 mg pills per day (730 per year).						
Price	1,858	1,259	1,714	1,294	1,536	R, NA
MMW	3.5	3.8	5	4	14	--
MIPC	1.5	1.5	0.9	2	2	--
**Azilsartan medoxonil,** 80 mg once a day (365 pills per year).						
Price	R, NA	--	R, NA	--	1,026	R, NA
MMW	--	--	--	--	10	--
MIPC	--	--	--	--	1.2	--
**Belatacept,** 10 mg per Kg per treatment. A person weighing up to 50 Kg needs 2 vials per treatment, and 16 treatments per year.						
Price	42,508	3,293	R, NA	--	R, NA	--
MMW	81	10	--	--	--	--
MIPC	35	4	--	--	--	--
**Belimumab,** 10 mg/Kg at 2-week interval for the first 3 doses and 4 weeks intervals thereafter. For a total of 15 treatments per year.						
Price	N I	20,995	7,725	R, NA	NI	NR, GSK says it is A
MMW	--	64	20	--	--	--
MIPC	--	23	6	--	--	--
**Crizotinib**	--	R, NA		--	--	--
**Indacaterol,** 75 μg once a day						
Price						
150μg	798	--	435	878	--	NI
300μg	844	--	809	NA	--	NI
MMW						
150μg	1.5	--	1.1	3.	--	--
300μg	1.6	--	2.5	NA	--	--
MIPC						
150 μg	0.6	--	0.5	1.3	--	--
300μg	0.7	--	0.6	NA	--	--
**Ipilimumab,** 3mg/kg IV infusion over 90 minutes every 3 weeks for 4 cycles						
Price	175,697	100,189	96,212	--	--	NI
MMW	336	305	249	--	--	NI
MIPC	143	107	74	--	--	NI
**Linagliptin,** 5 mg once a day						
Price	1,012	--	--	--	1,120	NI
MMW	1.9	--	--	--	10	NI
MIPC	0.8	--	--	--	1.3	NI
**Pasireotide,** 600 or 900μg twice a day						
Price						
600μg	143,309	R, A?	--	--	88,061	--
900μg	164,799	--	--	--	99,413	--
MMW						
600μg	274	--	--	--	793	--
900μg	315	--	--	--	896	--
MIPC						
600μg	117	--	--	--	113	--
900μg	134	--	--	--	116	--
**Pertuzumab,** Initial dose 840mg as a 60-minute IV infusion, followed every 3 weeks thereafter by 420mg as a 30 to 60 minute IV infusion						
Price	**--**	58,979	**--**	**--**	73.713	R, A?
MMW	**--**	179	**--**	**--**	644	**--**
MIPC	**--**	75	**--**	**--**	85	**--**
**Regorafenib,** 4 tablets a day, 21 days of 28-day cycle						
Price	19,584	NI	**--**	**--**	**--**	**--**
MMW	37	--	**--**	**--**	**--**	**--**
MIPC	16	--	**--**	**--**	**--**	**--**
**Rilpivirine,** For HIV-1 infection in adults who have never taken HIV therapy	R, A?	NR, NA	NR, A?	--	R, A?	--
**Rivaroxavan,** 10 mg once a day (Knee = 12 days; Hip = 35 days)						
Price Knee	162	42.6	55.6	170	59	60
MMW	0.3	0.1	0.1	0.6	1	0.2
MIPC	0.1	0.1	0.04	0.3	0.1	0.1
Price Hip	476	124	162	496	171	160
MMW	0.9	0.4	0.4	1.6	2	1
MIPC	0.4	0.1	0.1	0.8	0.2	0.3
**Roflumilast,** One tablet 500 mcg per day						
Price	--	993	--	--	--	--
MMW	--	3	--	--	--	--
MIPC	--	1	--	--	--	--
**Taliglucerasa alfa,** 60 units/kg IV every 2 weeks, 26 treatments per year						
Price	--	--	266,960	--	--	--
MMW	--	--	584	--	--	--
MIPC	--	--	203	--	--	--
**Telaprevir,** 1125 mg twice a day, for 12 weeks						
Price	52,061	44,554	**--**	**--**	**--**	**--**
MMW	99	135	**--**	**--**	**--**	**--**
MIPC	42	48	**--**	**--**	**--**	**--**
**Teriflunomide**	**--**	**--**	R, A?	**--**	R, A?	**--**
**Ticagrelor,** 90 mg twice a day						
Price	2,681	1,407	--	--	1,879	--
MMW	5	4	--	--	17	--
MIPC	2	1.5	--	--	1.8	--
**Tofacitinib,** 5mgr twice a day						
Price	45,252	NR, NA	N I	13,504	18,308	R, A?
MMW	86.5	--	--	44	165	--
MIPC	37	--	--	21	22	--
**Vandetanib,** 300 mg once a day						
Price	213,618	117,848	--	--	NR, A?	--
MMW	408	358	--	--	--	--
MIPC	174	126	--	--	--	--

Information regarding the methodology to determine the MMW and MIPC can be found in Homedes and Ugalde.^10^ In Ecuador there was information only for Indacaterol, and to simplify the table we have not included the information and is available from corresponding author.

^a^ For cells with no information, no clinical trials were conducted in the country or the product is not available. A = Available, A? = could not confirm if the product was available in the specific country, NA = Not Available; NI = No information available: R = registered: NR = Not registered

^b^In Brazil, Anvisa publishes a list of maximum prices for the consumer that does not include taxes. The tax rate varies by state from 0–19%. We have included the maximum tax rate to the Anvisa prices.

^c^ All GDP data are reported in PPP which are in US dollars and based on Atlas methodology used by the IMF and the World Bank. The Argentina GDP per capita is based on data officially reported by the National Statistics and Censuses Institute of Argentina that do not estimate the PPP. The International Monetary Fund (IMF) has called on Argentina to adopt measures to address the quality of official GDP and consumer price index data. More information available in http://data.worldbank.org/indicator/NY.GDP.PCAP.PP.KD

Prices varied widely by country, both in absolute and in relative terms. Argentina had the highest absolute price for many of the drugs included in this study (aflibercept, apixaban, belatacept, ipilimumab, pasireotide, telaprevir, ticaglecor, tofacitinib, vandetanib), on occasion even doubling the second highest price (aflibercept, belatacept, tofacitinib). Brazil had the lowest prices for apixaban, belatacept, pertuzumab, rivoraxavan and ticaglecor, but the price of belimumab was more than twice that in Chile. The price of belimumab, indacaterol and ipilimumab was lowest in Chile; Colombia had the lowest price for aflibercept and tofacitinib, and the highest for rivoraxavan.

We could not find any relationship between prices and the GDP per capita or the minimum wages in these countries. In Brazil aflibercept costs US$15,259 per course of treatment; in Argentina, which has a slightly lower GDP per capita than Brazil, the course of treatment of the same medication is US$30,410. Brazilians pay 46 times the monthly minimum wage and Argentineans 58, unaffordable in both countries but considerably more in Argentina. If we compare aflibercept in Colombia and Chile, which has a considerably higher GDP per capita than Colombia, the drug is slightly cheaper in Colombia, but in Colombia patients have to pay twice as many monthly minimum wages as Chileans. In the case of indacaterol, in Colombia the cost of the drug is twice that in Chile, and the same is true for rivaroxavan. Many other significant price and monthly minimum wages differences can be found in Table 4.

## Discussion

The pharmaceutical industry had commercialized about two thirds of the NMEs approved by the FDA in the Latin American countries where they had been tested, within two years, but only a handful offered some advantage over existing treatments for specific groups of patients. Despite large price differentials across countries, all NMEs but one were being sold at highly unaffordable prices [11].

Fojo et al. [10], documented that the median gain in progression-free and overall survival offered by therapies for solid tumors approved by the FDA between 2002 and 2014 (N = 71) were 2.5 and 2.1 months, respectively. Subsequently, the authors used standards similar to those developed by four disease-specific groups of ASCO’s Research Committee to determine the clinical relevance, in terms of overall survival and/or quality of life, of the 71 NMEs approved by the FDA during the 12 year period. Although the standards were recognized as modest, the authors concluded that only 30 NMEs (or 42%) provided “clinically meaningful improvements.”

We have not found any explanation in the literature for the significant price differentials of the same NMEs across the Latin American countries. Exploring the reasons for the differences would require a detailed analysis of all the drug pricing components in each country, including the manufacturer’s sale price, transportation costs, importation tariffs, the margin of benefits for distributors and dispensers, sales taxes and others. This analysis could guide governmental decisions to make products more affordable, but it is unlikely to explain the wide price-differentials that we have observed in the region. Moreover, our data do not support the hypothesis that pharmaceutical manufacturers are abiding by WHO recommendations and setting prices according to the wealth of countries (differential pricing) [11]. Why the same drug requires a higher financial burden for Colombians than Brazilians, Mexicans or other Latin Americans needs to be clarified. More collaboration among pharmaceutical policy makers and procurement experts across countries could lead to improved pricing structures for the region.

In Latin America, patients and patient groups—often financially supported by the innovative pharmaceutical companies—are increasingly using their Constitutional right to health to sue the governments [40–44] to gain access to the newest treatments that had not been included in the national formularies [45–47]. It has been documented that judges base their decisions on individual needs instead of societal priorities; if this trend continues, health care systems will be severely strained and many could go bankrupt [46–53]. This practice, known as judicialization, may have the undesirable health effect of exposing patients to NMEs that according to independent drug bulletins should not be used.

Given the approval process of the FDA and EMA [54–62], which tend to approve all NMEs without ensuring that they are more effective and/or safer than existing treatments, the Latin American regulatory agencies could consider delinking their drug approval decisions from those agencies and instead use the advice of independent organizations that tend to provide more accurate assessments of the therapeutic value of the NMEs. Because of the dearth of true innovation, delaying the approval of NMEs until independent reports are available will not result in detrimental health effects for the residents in these countries. Exceptions could be made for true breakthrough NMEs. Given the relative importance of these pharmaceutical markets, such a change would not have a significant impact on the economic performance of the industry.

The fact that a large proportion of NMEs failed to add therapeutic value to existing treatments and had significant side effects leads us to conclude that the patients enrolled in the experimental arm of the clinical trial were in fact worst off than if they had received the standard treatment. Similarly, the patients included in the control arm, except those who received the best available treatment, were also incurring unnecessary risks, especially if they were enrolled in a placebo-controlled or non-inferiority trial. Some of these risks could potentially have been avoided if research sponsors had conducted a more in-depth analysis of the results of pre-clinical studies and of earlier phases of the CTs [63, 64] and if the NMEs were always tested against the best available treatment. To consciously expose research participants to unnecessary risks would translate into a violation of the ethical principle of beneficence.

According to article 20 of the Helsinki Declaration, vulnerable populations should not be subjects in clinical trials when the products can be tested in non-vulnerable populations. All products included in this study, except bedaquiline, which is used in the treatment of multidrug-resistant tuberculosis, could have been tested in non-vulnerable populations. In Latin America, most subjects who participate in clinical trials tend to be of low socioeconomic status, are often medically illiterate, and according to some authors should be considered vulnerable [65–67].

If the 26 products included in our study, for which we obtained efficacy and safety information from independent sources, are a representative sample of the efficacy and safety of drugs that are tested in Latin America, there are several questions that need to be pondered: (1) Were all the clinical trials necessary? (2) Did the CTs have to be conducted in the vulnerable populations of Latin America? (3) Were the risks and benefits of participating in research being equally distributed in the population, as required by the ethical principle of justice?

Study Limitations. The FDA reviews included the clinical trials with the NMEs but did not always specify which clinical trials were used to approve the NME. As a result in a few cases we could have included countries where non-pivotal clinical trials were conducted. The information on medicine prices was collected in August-September 2014, and the information on GDP and Minimum wage dates from 2013 or 2014. Moreover, the countries included in our countries are highly inequitable; therefore the population in the lowest income deciles would have harder difficulties accessing the NMEs than we have reported in this study.

## Conclusion

Three of the five products that the independent drug bulletins classified as offering some advantage over existing treatments for some patients were commercialized in the Latin American countries where tested (ipilimumab, pasireotide, telaprevir), but their cost, above US$44.000 per year or per treatment, made them unaffordable to the majority of the Latin American population.

The outsourcing of clinical trials to Latin America may have produced some financial benefits to the pharmaceutical corporations but may also financially strain public budgets while exposing test subjects and those who access the NMEs to health risks.

Latin American regulatory agencies should be very cautious in adopting the commercialization decisions of the FDA or of the regulatory agencies of other high sanitary surveillance countries, and include the advice of independent research groups in their regulatory decisions.

Since the large majority of CTs included in this review have failed to demonstrate new therapeutic value, and instead have largely resulted in the commercialization of drugs that independent drugs bulletins consider to be less safe than available therapies, Latin American governments, regulatory agencies and research ethics committees should be very vigilant when authorizing clinical trials, at least until the innovative pharmaceutical companies reverse the current research and development (R&D) model. It is unethical to expose subjects to high health risks, particularly in Latin America where most of the subjects are vulnerable, when the potential benefits for them or for their country are limited.

In addition, by approving the implementation of the CTs of NMEs, the countries risk having to purchase very expensive products, endangering the budget of the ministries of health without improving the health of the patients.

It will be useful to analyze the reasons for the differences in the price of the drugs across the different countries of the region. While the answer might be multifactorial, we hypothesize that a major contributor is that pharmaceutical firms charge whatever they consider the country is willing to pay.
